# Statin (3-hydroxy-3-methylglutaryl-coenzyme A reductase inhibitor)-based therapy for hepatitis C virus (HCV) infection-related diseases in the era of direct-acting antiviral agents

**DOI:** 10.12688/f1000research.7970.3

**Published:** 2017-01-25

**Authors:** Sara Sobhy Kishta, Sobhy Ahmed Kishta, Reem El-Shenawy

**Affiliations:** 1 Medical Biotechnology Lab, Microbial Biotechnology Department, National Research Center, Egypt, Cairo, Egypt; 2 Department of Surgery, Theodor Bilharz Research Institute, Giza, Egypt

**Keywords:** Hepatitis C virus (HCV) infection, HCV infection-related diseases, statins, replicon system, human hepatocyte-like cells from human induced pluripotent stem cells, direct-acting antiviral agents (DAAs)

## Abstract

Recent improvements have been made in the treatment of hepatitis C virus (HCV) infection with the introduction of direct-acting antiviral agents (DAAs). However, despite successful viral clearance, many patients continue to have HCV-related disease progression. Therefore, new treatments must be developed to achieve viral clearance and prevent the risk of HCV-related diseases. In particular, the use of pitavastatin together with DAAs may improve the antiviral efficacy as well as decrease the progression of liver fibrosis and the incidence of HCV-related hepatocellular carcinoma. To investigate the management methods for HCV-related diseases using pitavastatin and DAAs, clinical trials should be undertaken. However, concerns have been raised about potential drug interactions between statins and DAAs. Therefore, pre-clinical trials using a replicon system, human hepatocyte-like cells, human neurons and human cardiomyocytes from human-induced pluripotent stem cells should be conducted. Based on these pre-clinical trials, an optimal direct-acting antiviral agent could be selected for combination with pitavastatin and DAAs. Following the pre-clinical trial, the combination of pitavastatin and the optimal direct-acting antiviral agent should be compared to other combinations of DAAs ( *e.g.*, sofosbuvir and velpatasvir) according to the antiviral effect on HCV infection, HCV-related diseases and cost-effectiveness.

## Introduction

Hepatitis C virus (HCV) infection is an important public health problem worldwide, as many HCV-infected patients develop liver cirrhosis and/or hepatocellular carcinoma (HCC) ^1^.

Recent improvements in the treatment of HCV infection have focused on the use of direct-acting antiviral agents (DAAs) ^1^. However, despite successful viral clearance, many patients with advanced fibrosis continue to have HCV-related disease progression ^2, 3^. Therefore, new treatments must be developed to achieve viral clearance and prevent the risk of HCV-related diseases.

Statins (inhibitors of 3-hydroxy-3-methylglutaryl-coenzyme A reductase) have been proposed as a new candidate for the treatment of HCV infection. According to previously conducted clinical studies using statins in HCV-infected patients ^4– 8^, the antiviral effect of statins on HCV infection depends on the type of statin used. Among various statins, pitavastatin showed the highest antiviral efficacy against HCV genotype 1b infection *in vitro* ^9^. Therefore, pitavastatin represents a novel candidate for the treatment of HCV infection.

## Clinical trials for pitavastatin against HCV infection

After performing a search of the PubMed database, we identified three clinical trials using pitavastatin for the treatment of HCV infection ^7, 8, 10^.

When Shimada *et al.* ^7^ considered two reports published in 2010 investigating the antiviral efficacy of pitavastatin against HCV infection *in vitro* ^9, 11^, they conducted a randomized controlled trial ^7^. The proof-of-concept studies demonstrated the antiviral efficacy and safety of pitavastatin against HCV infection using a replicon system and human hepatocyte-like cells from human induced pluripotent stem cells (hiPSCs) ^9, 11^, and these effects of pitavastatin were confirmed in the randomized controlled trial ^7, 12^. Indeed, this series of studies ^7, 9, 11^ would be the first to report the clinical applications of hiPSCs ^12^. After the clinical trial performed by Shimada *et al.* ^7^, the antiviral efficacy and safety of pitavastatin against HCV infection were further confirmed in two clinical studies ^8, 10^. Thus, investigating the antiviral efficacy and safety of pitavastatin against HCV infection using a replicon system and human hepatocyte-like cells from hiPSCs ^9, 11^ constitutes a rational approach to discover new drugs and/or new therapeutic methods.

## Clinical trials to investigate the management methods of HCV-related diseases in the era of DAAs

Butt *et al.* ^13^ showed that statin use was associated with improved antiviral efficacy as well as decreased progression of liver fibrosis and a reduced incidence of HCC among a large cohort of HCV-positive veterans. Furthermore, the use of statins among patients with HCV and compensated cirrhosis (n=40,512) was associated with a more than 40% lower risk of cirrhosis decomposition and death ^14^. Moreover, statin users showed a significant reduction in the incidence of HCC ^15^. In addition, pitavastatin showed anti-cancer effects against human hematoma cell lines ^16– 18^.

DAAs in combination with statins have been shown to generate increased antiviral efficacy against HCV infection ^19^. However, concerns have been raised about the drug interactions between various statins and DAAs ^20^. For instance, simvastatin and lovastatin should be avoided in patients with HCV infection who are using boceprevir or telaprevir as a DAA ^21^. Atorvastatin should be avoided in patients with HCV infection who are using telaprevir ^21^, and pravastatin plus boceprevir may also pose risks ^21^. Although rosuvastatin could be considered for use in combination with telaprevir and boceprevir ^21^, the drug interactions between pitavastatin and DAAs remain unknown.

Furthermore, according to our search of the PubMed database and UMIN Clinical Trials Registry System ( http://www.umin.ac.jp/icdr/index.html), no clinical trial has been conducted for the combination of statins and DAAs. Therefore, although there is likely only a minimal additive benefit for viral clearance using statins, as new combinations of DAA (sofosbuvir and velpatasvir) therapy have shown sustained virologic response (SVR) rates above 95% ^22^, conducting a clinical trial for the combination of pitavastatin and DAAs may be meaningful to investigate management methods to prevent fibrosis and cirrhosis or the development of HCC and other HCV-related diseases in the era of DAAs. However, in pre-clinical trials, the antiviral effects of the combination of pitavastatin and DAAs should be evaluated using a replicon system ^9^. Furthermore, hepatotoxicities should also be evaluated for the combination of pitavastatin and DAAs using human hepatocyte-like cells from hiPSCs ^11^. Using a replicon system and human hepatocyte-like cells from hiPSCs in a pre-clinical trial ^9, 11^, an optimal direct-acting antiviral agent could be selected for use in the combination of pitavastatin and DAAs. After the pre-clinical trial, the combination of pitavastatin and the optimal direct-acting antiviral agent should be compared with other DAA combinations ( *e.g.*, sofosbuvir and velpatasvir) according to their antiviral efficacy against HCV infection and prevention of HCV-related diseases. Furthermore, because the cost for DAA combination treatment is very high ($83,000 to $153,000 per course of treatment) ^22^, the new and effective hepatitis C treatments seem beyond the reach of low- and middle-income countries ^23^. However, the cost ($0.79 to $2.59 per day) of pitavastatin (2mg) ^7^ is low ( http://www.pharmacychecker.com/generic/price-comparison/pitavastatin/2+mg/). Therefore, the above-mentioned comparison should also be investigated in light of cost-effectiveness.

On the other hand, a replicon system in hepatocellular cells is not likely to duplicate the full gamut of potential side effects seen in patients with HCV infection receiving pitavastatin and DAAs. The risks (potential side effects) that will not be revealed in a pre-clinical trial using this system should be acknowledged. Therefore, in order to identify the risk (potential side effects) that will not be revealed in a pre-clinical trial using this system, the evaluations using human neurons and human cardiomyocytes from hiPSCs ^24, 25^ should also be done in pre-clinical trials.

In conclusion, a pre-clinical trial investigating the combination of pitavastatin and DAAs against HCV infection using a replicon system, human hepatocyte-like cells, human neurons and human cardiomyocytes from hiPSCs ^9, 11, 24, 25^ represents a rational approach for discovering a new therapeutic method.
