# Supplementary material for: Statin (3-hydroxy-3-methylglutaryl-coenzyme A reductase inhibitor)-based therapy for hepatitis C virus (HCV) infection-related diseases in the era of direct-acting antiviral agents
Source: F1000Res. 2017 Jan 25;5:223. Originally published 2016 Feb 26. [Version 3] doi: 10.12688/f1000research.7970.3 (PMC4988296; doi:10.12688/f1000research.7970.3)
Supplement: Peer review history

# Peer review: Referee response for version 2

**DOI:** 10.5256/f1000research.9843.r15693

**Published:** 2016-08-15

**Authors:** Joel D Baines

**Affiliations:** 1 LSU School of Veterinary Medicine (SVM), Louisiana State University, Baton Rouge, LA, USA

The revisions address my concerns about the preclinical studies.

I have read this submission. I believe that I have an appropriate level of expertise to confirm that it is of an acceptable scientific standard.

# Peer review: Referee response for version 1

**DOI:** 10.5256/f1000research.8578.r13945

**Published:** 2016-06-07

**Authors:** Joel D Baines

**Affiliations:** 1 LSU School of Veterinary Medicine (SVM), Louisiana State University, Baton Rouge, LA, USA

This article would be improved with statements quantifying how common progression to disease occurs in patients treated with anti-HCV compounds in the absence of other drugs. The main point of the article, that there should be pre-clinical trials to test for efficacy and toxicity of dual therapy with Pitavastatin are valid.  However, a replicon system in hepatocellular cells is not likely to duplicate the full gamut of potential side effects seen in humans receiving pitavastatin and anti-HCV drugs.  Although it is an important place to start, the risk that complications will not be revealed in a preclinical trial using this system should be acknowledged.

I have read this submission. I believe that I have an appropriate level of expertise to confirm that it is of an acceptable scientific standard, however I have significant reservations, as outlined above.

# Response

**Published:** 2016-06-28

**Authors:** Sara Kishta

**Affiliations:** National Research Center, Egypt, Egypt

First of all, thank you very much for your suggestions. We think that your comments were very helpful. We agree with your suggestions. If you agree with our revisions, we are very happy.

 1)  According to your suggestions, we added the following sentences to our text (please see, last paragraph and conclusion).

On the other hand, a replicon system in hepatocellular cells is not likely to duplicate the full gamut of potential side effects seen in patients with HCV infection receiving pitavastatin and DAAs. The risks (potential side effects) that will not be revealed in a pre-clinical trial using this system should be acknowledged. Therefore, in order to identify the risk (potential side effects) that will not be revealed in a pre-clinical trial using this system, the evaluations using human neurons and human cardiomyocytes from hiPSCs ^24, 25^ should also be done in pre- clinical trials .

 In conclusion, a pre-clinical trial investigating the combination of pitavastatin and DAAs against HCV infection using a replicon system, human hepatocyte-like cells, human neurons and human cardiomyocytes from hiPSCs ^9,11,24,25^ represents a rational approach for discovering a new therapeutic method.

 2)  We revised our abstract. 

 pre-clinical trials using a replicon system , human hepatocyte-like cells, human neurons and human cardiomyocytes from human-induced pluripotent stem cells should be conducted.

 3) We added the new references (No. 24 and 25) in the reference section.

# Peer review: Referee response for version 1

**DOI:** 10.5256/f1000research.8578.r13680

**Published:** 2016-05-11

**Authors:** Susana N. Asin

**Affiliations:** 1 White River Junction, Veterans Affairs Medical Center, White River Junction, VT, USA

Recent improvements in treatment strategies for Hepatitis C virus infected patients have been successful at reaching sustained virological responses yet did not prevent liver fibrosis and progression to hepatocellular carcinoma.

The idea proposed in this paper that new treatments must be developed to achieve viral clearance while preventing disease progression although meritorious is not original and should be complemented with further understanding of mechanisms underlying hepatitis C disease progression in the presence of sustained virological response (i.e. absence/ or low level of viral replication).

The authors stated that pitasvatin represents a novel candidate to treat HCV infection yet the rationale for selecting this statin, as the likely candidate is not clearly justified. The authors based their selection on data from a retrospective analysis demonstrating no significant difference in sustained virological response rate per protocol analysis between patients treated with PEG-IFN/ribavirin in the presence or absence of pitavastatin. Findings from a second prospective trial demonstrates the safety of pitavastatin in combination with Peg IFN plus ribavirin although the decrease in HCV RNA was only significant at 2 (4 and 12 weeks) of 6 evaluated time points of treatment. The third trial used pitavastin in combination with eicosapentaenoic acid and identified this combination therapy as predictive of sustained virological response in multifactorial analysis only after genetic variation in IL28B was excluded from these factors. Thus the authors could have presented a better rationale for the selection of this statin.

An additional point of discussion that should have been included in this paper would have been the evidence that statin use decreased progression to liver fibrosis and hepatocellular carcinoma yet this effect was independent of having attained a sustained virological response. These findings raised the possibility that the statins-mediated  delay in  liver fibrosis are related to their immunomodulatory rather than their antiviral effects. This assumption is supported by findings demonstrating little or no effect of statins use on HCV replication.

I have read this submission. I believe that I have an appropriate level of expertise to confirm that it is of an acceptable scientific standard.
